# How Did People Cope During the COVID-19 Pandemic? A Structural Topic Modelling Analysis of Free-Text Data From 11,000 United Kingdom Adults

**DOI:** 10.3389/fpsyg.2022.810655

**Published:** 2022-06-06

**Authors:** Liam Wright, Meg Fluharty, Andrew Steptoe, Daisy Fancourt

**Affiliations:** ^1^Institute of Education, University College London, London, United Kingdom; ^2^Department of Behavioural Science and Health, University College London, London, United Kingdom

**Keywords:** COVID-19, mental health, coping (C), free-text analysis, structural topic modeling, text mining

## Abstract

**Background:**

The COVID-19 pandemic has had substantial impacts on lives across the globe. Job losses have been widespread, and individuals have experienced significant restrictions on their usual activities, including extended isolation from family and friends. While studies suggest population mental health worsened from before the pandemic, not all individuals appear to have experienced poorer mental health. This raises the question of *how* people managed to cope during the pandemic.

**Methods:**

To understand the coping strategies individuals employed during the COVID-19 pandemic, we used structural topic modelling, a text mining technique, to extract themes from free-text data on coping from over 11,000 UK adults, collected between 14 October and 26 November 2020.

**Results:**

We identified 16 topics. The most discussed coping strategy was ‘thinking positively’ and involved themes of gratefulness and positivity. Other strategies included engaging in activities and hobbies (such as doing DIY, exercising, walking and spending time in nature), keeping routines, and focusing on one day at a time. Some participants reported more avoidant coping strategies, such as drinking alcohol and binge eating. Coping strategies varied by respondent characteristics including age, personality traits and sociodemographic characteristics and some coping strategies, such as engaging in creative activities, were associated with more positive lockdown experiences.

**Conclusion:**

A variety of coping strategies were employed by individuals during the COVID-19 pandemic. The coping strategy an individual adopted was related to their overall lockdown experiences. This may be useful for helping individuals prepare for future lockdowns or other events resulting in self-isolation.

## Introduction

The COVID-19 pandemic subjected people worldwide to a range of adversities, from isolation at home to loneliness, worries about and experiences of catching the virus, troubles with finances, difficulties acquiring basic needs, and boredom (Chandola et al., 2020; Wright et al., 2020; Brodeur et al., 2021; ONS, 2021). While some of these experiences have also been reported during previous epidemics (Brooks et al., 2020), the COVID-19 pandemic was unprecedented in its global size, transmissibility, and uncertain timeframe. As a result, there were serious concerns that people would be unable to cope and there would be a substantial rise in mental illness, self-harm and suicide globally (Holmes et al., 2020; Mahase, 2020). To a certain extent, this was borne out, with data showing rises in depression and anxiety at the start of the pandemic in many countries around the world (Banks and Xu, 2020; Schippers, 2020; Pierce et al., 2021). However, the COVID-19 pandemic also highlighted resilience amongst many groups, manifested as either levels of anxiety, depression and life satisfaction returning relatively quickly to pre-pandemic levels (Fancourt et al., 2021; Pierce et al., 2021), or with certain groups such as older adults only experiencing small changes to their mental health (Fancourt et al., 2021), or not showing any signs of worsened mental health at all (Saunders et al., 2021). This raises the question of *how* people managed to cope during the pandemic.

How people cope is an important factor underlying the relationship between experiencing stressors and subsequent mental health. Coping is generally defined as the cognitive and behavioural efforts that are used to manage stress (Lazarus and Folkman, 1991). There is much debate as to whether certain coping strategies are more beneficial than others and in what contexts. For example, strategies that aim to reduce and resolve stressors may be more effective in supporting mental health (Taylor and Stanton, 2007), but avoidant strategies may be helpful in reducing short-term stress (Taylor and Stanton, 2007). While avoidant strategies may have some benefits, they can also lead to more harm as no direct actions are taken to reduce the stressor, potentially resulting in feelings of helplessness or self-blame (Suls and Fletcher, 1985). Numerous sociodemographic, personality, and social factors are known to influence how people cope with stress (Bolger and Zuckerman, 1995; Christensen et al., 2006). For example, personality type can influence the severity of the stressor experience by facilitating or constraining use of coping strategies (Bolger and Zuckerman, 1995; Connor-Smith and Flachsbart, 2007). Additionally, effects of personality on coping are facilitated by their consequences for level of engagement with stressors, and similarly, approach to rewards (Connor-Smith and Flachsbart, 2007; Leszko et al., 2020).

A number of studies have examined coping during the COVID-19 pandemic, using quantitative (Park et al., 2020; Agha, 2021; Fluharty and Fancourt, 2021), qualitative (Ogueji et al., 2021; Sarah et al., 2021), and mixed methods approaches (Chew et al., 2020; Dewa et al., 2021). Quantitative studies have examined the association between coping strategies and the level and trajectories of symptoms of poor mental health during the pandemic (Agha, 2021; Fluharty et al., 2021). For example, coping strategies involving withdrawal, avoidance and substance use to evade the source of stress have been shown to partially mediate trajectories of depression and anxiety during COVID-19 (Freyhofer et al., 2021, p. 19). Further, a study examining predictors of coping found COVID-19 specific experiences contributed to choice of coping strategy (Fluharty and Fancourt, 2021). For instance, experiencing financial adversity (such as job loss and major cut in income) was associated with problem-focused, emotion-focused, and avoidant coping. Additionally, two qualitative studies collected free text responses on how people were coping during the COVID-19 pandemic (Ogueji et al., 2021; Sarah et al., 2021). Both studies found the most common strategies employed were centred around socially-supported coping. However, these studies had small sample sizes and were both recruited over social media, which may have biased the sample towards this result. Further, the studies were narrative in nature and did not compare coping strategies across sociodemographic groups or in a formal manner to peoples’ lockdown experiences.

There has also been a methodological challenge with the studies on coping during COVID-19 carried out so far. Existing quantitative studies have typically relied on closed-form responses to survey items (e.g., Likert responses). An issue with this approach is that responses are restricted to those that the researcher has thought of in advance – an issue that is particularly salient given the novelty of the COVID-19 pandemic. While qualitative approaches allow for more flexibility in responses, the small sample sizes typical of qualitative studies restrict the questions that can be asked of the data – specifically, those that statistically relate individual characteristics and circumstances to topics raised.

Text-mining methods offer the benefits of quantitative and qualitative approaches, enabling the extraction of themes from large-scale free-text data that can be summarised numerically and related to participant characteristics (e.g., age, sex, and personality traits) using standard statistical methods. To our knowledge, two studies have used text-mining methods to analyse free-text survey data on coping during the COVID-19 pandemic in the United Kingdom (Rogers et al., 2020; Hampshire et al., 2021). Both found that visiting nature and green space, keeping active, and using videoconferencing to keep in touch with family and friends were common strategies employed to improve wellbeing. However, both studies used data from the first months of the pandemic, when the situation was relatively novel and social isolation had not been extended for long. Therefore, this study aimed to explore the breadth of coping strategies adopted over a longer period of the pandemic, and how these strategies related to participants’ demographic, socioeconomic and personality characteristics and to their lockdown experiences. To achieve this, we used structural topic modelling (STM; Roberts et al., 2014) – a text-mining technique – and free-text data from 11,000 United Kingdom adults that was collected seven months after lockdown was first introduced in the United Kingdom.

## Materials and Methods

### Participants

We used data from the COVID-19 Social Study; a large panel study of the psychological and social experiences of over 70,000 adults (aged 18+) in the United Kingdom during the COVID-19 pandemic. The study commenced on 21 March 2020 and involved online weekly data collection for 22 weeks with monthly data collection thereafter. The study is not a random sample and therefore is not representative of the United Kingdom population, but it does contain a heterogeneous set of individuals. Participants were recruited in three ways. First, convenience sampling was used, including promoting the study through existing networks and mailing lists (including large databases of adults who had previously consented to be involved in health research across the United Kingdom), print and digital media coverage, and social media. Second, more targeted recruitment was undertaken focusing on groups who were anticipated to be less likely to take part in the research *via* our first strategy, including (i) individuals from a low-income background, (ii) individuals with no or few educational qualifications, and (iii) individuals who were unemployed. Third, the study was promoted *via* partnerships with third sector organisations to vulnerable groups, including adults with pre-existing mental health conditions, older adults, carers, and people experiencing domestic violence or abuse. Full details on sampling, recruitment, data collection, data cleaning and sample demographics are available at https://doi.org/10.17605/OSF.IO/JM8RA. The study was approved by the UCL Research Ethics Committee (12467/005) and all participants gave informed consent.

A one-off free-text module was included in the survey between 14 October and 26 November 2020. Participants were asked to write responses to eight questions on their experiences during the pandemic and their expectations for the future. Here, we used responses to a single question: *What have been your methods for coping during the pandemic so far and which have been the most or least helpful?* (see Supplementary Table 1 for the full list of questions asked during the module). 30,950 individuals participated in the data collection containing this survey module (43.4% of participants with data collection by 26 November 2020). Responses to the free-text questions were optional. 12,536 participants recorded a response to the question on coping (40.5% of eligible participants). Of these, 11,073 (88.3%) provided a valid record, the definition of which is provided in a following section.

The period 14 October–26 November was seven months into the pandemic in the United Kingdom and overlapped with the beginning of the second wave of the virus. As such, participants could reflect on their experiences during a strict lockdown from March 2020, the relaxation of that lockdown over the summer of 2020, and the start of new restrictions being brought in for the second wave. Supplementary Figure 1 shows 7-day COVID-19 caseloads and confirmed deaths, along with the Oxford Policy Tracker, a numerical summary of policy stringency (Hale et al., 2020), across the study period. An overview of the key developments in the pandemic across the data collection period is provided in the Supplementary Information.

### Predictors of Topic Proportions

Structural topic modelling allows for inclusion of covariates in the estimation model, such that the estimated proportion of a free-text response devoted to a given topic can differ according to document metadata (e.g., characteristics of its author). To predict topic proportions, we included variables for age, sex, ethnicity, country of residence, education level, living arrangement, keyworker status, self-isolation status, diagnosed psychiatric condition, long-term physical health conditions, and Big-5 personality traits.

Country of residence (England, Scotland, Wales, Northern Ireland), sex (male, female), ethnicity (White, Non-White), age (modelled with basis splines [B-Splines] with four degrees of freedom (Perperoglou et al., 2019) to account for potential non-linear association), education level (GCSE or below, A-levels or equivalent, degree or above), and keyworker status (as working in health, social care or support sectors, or work involving in medicines or PPE production or distribution) were each measured at baseline interview. Long-term physical health conditions (0, 1, 2+) was measured using a multiple-choice question on medical conditions. Included conditions were high blood pressure, diabetes, heart disease, lung disease, cancer, any other clinically-diagnosed chronic physical health conditions, or any disability. Psychiatric diagnosis (yes, no) was measured with the same multiple choice question using items on clinically diagnosed depression, clinically diagnosed anxiety, and any other clinically diagnosed mental health problem. Both variables were collected at baseline interview. Self-isolation status was defined as staying at home at any point due to existing medical condition or being categorised as high risk. This variable was collected at data collections between 21 March 2020 and 04 July 2020.

Personality was measured at baseline interview using the Big Five Inventory (BFI-2; Soto and John, 2017), which measures personality on five domains and 15 facets: openness (intellectual curiosity, aesthetic sensitivity, and creative imagination), conscientiousness (organisation, productiveness, and responsibility), extraversion (sociability, assertiveness, and energy level), agreeableness (compassion, respectfulness, and trust) and neuroticism (anxiety, depression, and emotional volatility). Each item was scored on a 5-point scale (1 = “strongly disagree”, 5 = “strongly agree”). We used the sum Likert score for each domain (range 3–15). Higher scores indicate higher levels of the trait.

### Data Cleaning

We performed topic modelling using unigrams (single words). Free-text responses were cleaned using an iterative process. The main steps were as follows. Popular hyphenated words were collapsed into non-hyphenated form and spaces were removed between words that could have been hyphenated (e.g., “pre-pandemic” and “pre pandemic” became “prepandemic”). Punctuation mistakes (e.g., full stops between words) were replaced with whitespace unless the full stop denoted an initialism or a URL. Full stops were removed between initialisms – e.g., U.K. became UK – and “www.” was removed from URLs. Responses were tokenized into lower-case unigram form and “stop” words (common words such as “the” and “and”) were removed. Stop words were identified with the onix, SMART, and snowball dictionaries (Silge and Robinson, 2016), excluding 38 words that we deemed to be relevant to the current topic. We identified spelling mistakes with the hunspell spellchecker (Ooms, 2018), and amended these manually if they had FOUR or more occurrences, and replaced using the hunspell suggested word function otherwise. Where the algorithm provided multiple suggestions, the word with the highest frequency across responses was used. To reduce data sparsity, in the STM analysis, we further stemmed words using the Porter (1980) algorithm, dropped responses if they contained fewer than five words, and dropped words if they appeared in fewer than five responses (Banks et al., 2018). Data cleaning was carried out in R version 3.6.3 (R Core Team, 2020) using the tidyverse (Wickham et al., 2019), stringi (Gagolewski, 2020), qdap (Rinker, 2020), hunspell (Ooms, 2018), SnowballC (Bouchet-Valat, 2020), and tidytext (Silge and Robinson, 2016) packages.

### Data Analysis

We performed several quantitative analyses. First, as not all participants chose to provide a response, we ran a logistic regression model to explore the predictors of providing a free-text response. We used the variables defined above as predictor variables (to simplify interpretation, we converted age to categories; 18–29, 30–45, 46–59, 60+). Second, we used STM, implemented with the stm R package (Roberts et al., 2019), to extract topics from responses. STM treats documents as a probabilistic mixture of topics and topics as a probabilistic mixture of words. It is a “bag of words” approach that uses correlations between word frequencies within documents to define topics. As noted, STM allows for inclusion of covariates in the estimation model, and we included the variables defined above. There was only a small amount of item missingness (*n* = 113), so we used complete case data.

We ran STM models from 2 to 30 topics and selected the final models based on visual inspection of the semantic coherence and exclusivity of the topics and close reading of exemplar documents representative of each topic (documents with highest proportion of text estimated as belonging to a given topic). Semantic coherence measures the degree to which high probability words within a topic co-occur, while exclusivity measures the extent to that a topic’s high probability words have low probability for other topics. After selecting a final model, we carried out three further analyses. First, we decided upon narrative descriptions for the topics based on high probability words, high “FREX” words (a weighted measure of word frequency and exclusivity), and exemplar texts. Second, we ran multiply-adjusted linear regression models estimating whether topic proportions were related to author characteristics defined above (again categorising age into four groups to aid interpretability). For comparability with categorical variables, Big-5 personality trait variables were scaled such that a 1-unit change was equal to a 2 SD difference (Gelman, 2008). (Topic proportions were the dependent variables in these regressions.) Third, to explore which coping strategies may have been particularly effective, we used linear regression to examine whether topic proportions predicted lockdown experiences. Lockdown experiences were measured with three separate items on enjoying lockdown (How much have you enjoyed lockdown? 1. Not at all, 7. Very much), missing lockdown (Do you feel you will miss being in lockdown? 1. Not at all, 7. Very much), and feelings about future lockdowns (How do you feel about the prospect of any future lockdowns? 1. I would dread it, 7. I would really look forward to it). These variables were collected between 11 and 18 June 2020. We ran a separate regression for each lockdown experience variable, with each given variable regressed upon topic proportions added to the model simultaneously. We did not include intercepts in this regression, so coefficients can be interpreted as predicted means when all text is devoted to a specific topic. Individuals with item-missingness on the lockdown experience variables were dropped in this analysis (*n* = 2,203).

## Results

### Descriptive Statistics

A total of 11,073 individuals provided a valid free-text response. Descriptive statistics for respondents are displayed in Table 1, with figures for the total eligible sample also shown for comparison. There were some differences between those who provided a (valid) response and those that did not. Supplementary Figure 2 displays the results of logistic regression models exploring the predictors of providing a response. Responders were disproportionately female, of older age, more highly educated, more likely to live alone, and to have self-isolated than non-responders. They were also more open, conscientious, and extraverted, on average.

**TABLE 1:** Descriptive statistics.

	Variable	Eligible	% Missing	Answered	Valid
	n	30,950		12,536 (40.5%)	11,073 (35.78%)
Gender	Male	7,750 (25.14%)	0.39%	2,446 (19.61%)	2,038 (18.41%)
	Female	23,078 (74.86%)		10,027 (80.39%)	9,035 (81.59%)
Country	England	24,855 (80.31%)	0%	9,826 (78.38%)	8,683 (78.42%)
	Wales	3,989 (12.89%)		1,834 (14.63%)	1,614 (14.58%)
	Scotland	1,811 (5.85%)		764 (6.09%)	678 (6.12%)
	Northern Ireland	295 (0.95%)		112 (0.89%)	98 (0.89%)
Age Group	18–29	1,403 (4.53%)	0%	437 (3.49%)	381 (3.44%)
	30–45	6,255 (20.21%)		2,313 (18.45%)	2,060 (18.6%)
	46–59	10,045 (32.46%)		3,950 (31.51%)	3,476 (31.39%)
	60+	13,247 (42.8%)		5,836 (46.55%)	5,156 (46.56%)
Ethnicity	White	29,741 (96.4%)	0.31%	12,049 (96.49%)	10,688 (96.52%)
	Non-White	1,112 (3.6%)		438 (3.51%)	385 (3.48%)
Education	Degree or above	21,271 (68.73%)	0%	9,099 (72.58%)	8,157 (73.67%)
	A-Level	5,270 (17.03%)		1,933 (15.42%)	1,678 (15.15%)
	GCSE or below	4,409 (14.25%)		1,504 (12%)	1,238 (11.18%)
Keyworker	No	27,942 (90.28%)	0%	11,333 (90.4%)	10,019 (90.48%)
	Yes	3,008 (9.72%)		1,203 (9.6%)	1,054 (9.52%)
Living Arrangement	Not alone, no child	17,913 (57.88%)	0%	7,290 (58.15%)	6,440 (58.16%)
	Not alone, with child	6,334 (20.47%)		2,346 (18.71%)	2,053 (18.54%)
	Alone	6,703 (21.66%)		2,900 (23.13%)	2,580 (23.3%)
Psychiatric Diagnosis	No	26,081 (84.27%)	0%	10,532 (84.01%)	9,344 (84.39%)
	Yes	4,869 (15.73%)		2,004 (15.99%)	1,729 (15.61%)
Long-Term Conditions	0	17,432 (56.32%)	0%	6,821 (54.41%)	6,058 (54.71%)
	1	8,691 (28.08%)		3,653 (29.14%)	3,243 (29.29%)
	2+	4,827 (15.6%)		2,062 (16.45%)	1,772 (16%)
Self-Isolating	No	25,389 (82.03%)	0%	9,929 (79.2%)	8,790 (79.38%)
	Yes	5,561 (17.97%)		2,607 (20.8%)	2,283 (20.62%)
Big-5 Personality Traits	Openness	15.33 (3.26)	0%	15.82 (3.18)	15.87 (3.15)
	Conscientiousness	16.03 (2.91)	0%	16.24 (2.92)	16.26 (2.91)
	Extraversion	12.82 (4.27)	0%	13.29 (4.24)	13.31 (4.24)
	Agreeableness	15.55 (3.03)	0%	15.65 (3.03)	15.69 (3.02)
	Neuroticism	11.05 (4.26)	0%	11.03 (4.23)	11.01 (4.22)

**FIGURE 1:**
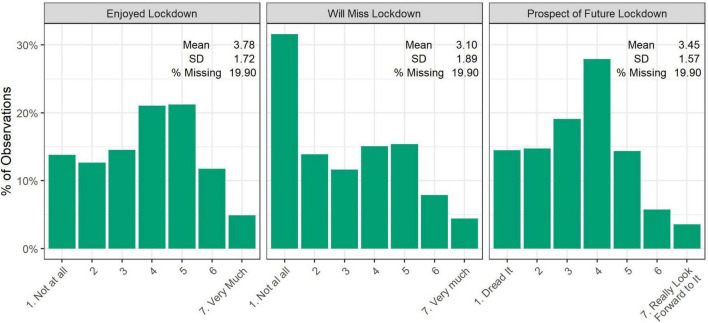
Descriptive statistics. Lockdown experience variables.

Descriptive statistics for the lockdown experience variables are displayed in Figure 1. Responses were varied, but more responses were recorded below the midpoint of the scales than above for each question. A higher mean response was given for the enjoyed lockdown question than for the other questions. The modal response to the will miss lockdown question was “not at all” (31.6%).

**FIGURE 2:**
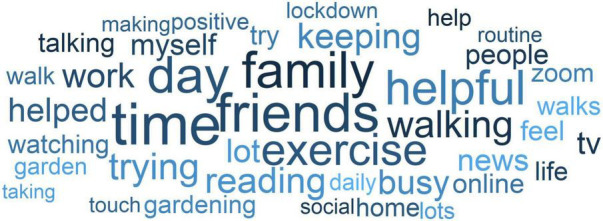
Word cloud. Forty most frequently used words across responses. Words sized according to number of responses they appear in.

A word cloud of the forty most frequently used words for each question is displayed in Figure 2. Many of the words refer to activities or time use (e.g., walking, reading, exercise, routine) or to social factors (e.g., friends, family, talking, zoom).

### Coping Strategies

We selected a 16 topic solution. Short descriptions are displayed in Table 2, along with exemplar quotes and topic titles that we use when plotting results. Topics are ordered according to the estimated proportion of text devoted to each topic. Correlations between the topic proportions are displayed in Supplementary Figures 3, 4.

**TABLE 2:** Topic descriptions.

Topic	Proportion	Short title	Description	Higher FREX words	Exemplar texts
1	8.22%	Thinking positively	Trying to see positives or count one’s fortunes. Recognising that the pandemic will pass	situat, try, posit, wors, rememb, bless, grate, focu, count, pass	“Trying to focus on what matters most and remember that all this will pass.” “When in any doubts creep in I just remember how many others are suffering far more or in situations worse then [sic] mine.”
2	8.14%	Engaging in harmful behaviours	Comfort eating, increasing alcohol intake and self-harm	drink, alcohol, method, cope, mechan, start, smoke, lockdown, lost, comfort	“Alcohol consumption increased during parts of this pandemic, which was not a sensible or healthy way of dealing with the stress. I am now over-eating as a coping mechanism, but again, I know that this is not a sensible method of coping with the stress and uncertainty of the situation. I have yet to find a method that is helpful.”
3	7.88%	Engaging in creative activities	Practicing arts, hobbies, and crafts	craft, bake, cook, lot, knit, sew, paint, creativ, medit, art	“Reading, writing, cookery, baking, crafts” “Meditation has helped with anxiety. Doing creative activities like painting-by-numbers, photography and reading have helped keep my mood higher.”
4	7.82%	Spending time in nature	Spending time in nature. In particular, going for walks.	dog, air, fresh, walk, countrysid, cycl, natur, park, mile, sane	“Walking in green spaces nearby has been very helpful. I’m lucky that I live in an area with plenty of nature around, so I have easy access to green spaces.” “Going for walks with my dog. Getting out in the fresh air and getting exercise with my dog is always an emotional boost”
5	7.77%	Consuming media	Listening to music and radio, watching TV and films.	tv, music, watch, film, listen, radio, game, seri, netflix, programm	“Listening to radio, music and distraction of TV dramas/lifestyle programs.”
6	7.34%	Taking one day at a time	Taking one day at a time and imposing structure.	dai, list, take, structur, hour, achiev, flat, morn, couch, set	“Having a structure to the day. Planning each day, being organised”
7	6.94%	Following the rules	Following guidelines and taking precautions when in public	govern, rule, wear, life, accept, normal, hand, ignor, death, awar	“Mostly following the advice of the government scientific advisors along with a common sense approach to safety” “To simply accept that by doing the right thing and following guidance is the only way in which we can affect the path of the disease. The more we do this, the shorter will be the disruption”
8	6.57%	Talking to family and friends	Talking with family by friends (often by video call).	talk, call, famili, friend, video, prayer, phonecal, facetim, messag, reach	“Speaking to family and friends by telephone, FaceTime and messaging.” “Talking to family and friends. Video calling grand children”
9	6.37%	Doing DIY and gardening	Gardening and “odd jobs” around the house	grow, project, decor, allot, veget, hous, summer, spring, winter, sort	“In the first lockdown I spent many hours gardening, growing my own vegetables. I found that very therapeutic and miss it now. I think the winter months will be far more difficult” “During the summer months I spent time doing jobs in the garden and house. Had a clear out around the house which was quite cathartic”
10	5.76%	Keeping busy	Keeping busy	busi, keep, touch, occupi, commun, volunt, husband, voluntari, vulner, sell	“Keeping busy. The house is spotless and I have been making toys to sell for charity.” “keeping myself occupied, but then I have been busy so that’s not really been an issue”
11	5.34%	Contacting others	Contact with others, especially over the internet or phone	phone, support, colleagu, bubbl, chat, close, grandkid, daughter, neighbour, meet	“zoom and telephone contacts with others” “Structure and scheduling appointments so I know I will have contact with other people. by skype or phone.”
12	4.79%	Keeping routines	Sticking with a routine, particularly with exercise.	usual, maintain, routin, restrict, cry, humour, limit, establish, lose, adapt	“Maintaining a regular routine even when working from home, and doing more home cooking. So I’m less healthy but more satisfied with my work/life balance.”
Topic	Proportion	Short title	Description	Higher FREX words	Exemplar texts
13	4.49%	Mixture of themes	Topic contains texts discussing disparate themes	down, moment, ahead, thank, futur, worri, head, slow, cbt, holidai	“Nothing really, just grin and bear it, there is little I can do to change things at the moment.” “My son is always able to make me laugh and I’m very thankful I live with my husband and son - I would struggle living alone during these times.”
14	4.23%	Doing online activities	Participating in activities online, such as classes and signing groups. Also contains texts discussing online supermarket shopping.	onlin, shop, cours, line, join, deliveri, visit, sing, class, pilat	“I have weekly Zoom sessions with my sisters and book group, plus monthly book discussions, I pay for live online story sessions, and interesting talks. I have also booked on to courses provided by my County Council library service. I have irregular Zoom meetings with my offspring, who live elsewhere” “Most helpful: the creation of sufficient delivery slots by food supermarkets. We are now having a weekly delivery and, although there are occasional shortages or substitutions, none of the missing items have been important.”
15	4.22%	Coping through exercise	Stating that exercise helps	help, feel, connect, allow, skill, find, improv, interact, other, exercis	“Exercise helps, but only when the anxiety is okay enough for me to be outside. Reopening pools, gyms and studios really helped as I could swim/dance and also socialise at the same time, which made me feel way less connected.”
16	4.13%	Avoiding the news	Cutting down on news and media consumption regarding the pandemic	neg, inform, avoid, media, focuss, follow, date, coverag, updat, overwhelm	“Most helpful is to sometimes switch off from the news/ social media. Peoples (sic) negative attitude in social media can be depressing, along with the news. To switch off for a while may be considered ignorant, but I feel it hugely helps mental health.”

The largest topic (Topic 1; 8.82% of text; Thinking positively) included individuals who had tried to see the positives in the situation, to remember that others were in relatively worse situations, and recognise that the pandemic would pass. Topic 6 (7.34%; Taking one day at a time) similarly, related to a general cognitive coping strategy, including text on individuals taking each day as it came and imposing structure on their time. This topic overlapped with Topic 12 (4.79%; Keeping routines), which related to people keeping routines, particularly with exercise. Similarly, Topic 10 (5.76%; Keeping busy) related to participants filling their time (“keeping busy”) in generally non-specific ways.

Most other topics related to spending time on specific activities. Topic 3 (7.88%; Engaging in creative activities) related to individuals engaging in arts, hobbies, or crafts as a way of coping. Topic 5 (7.77%; Consuming media) included text from participants who reported spending their time listening to music and radio or watching TV and films. Topic 4 (7.82%; Walking and spending time in nature) related to individuals who had used the opportunity to take long walks and get into nature, while Topic 15 (4.22%; Coping through exercise) included text from individuals who found exercise had a positive effect. Topic 8 (6.57%; Talking to family and friends) and Topic 11 (5.34%; Contacting others) including responses on keeping in contact with family, friends and colleagues, the latter referring to the use of online technologies in particular. Topic 9 (6.37%; Doing DIY and gardening) referred to individuals spending time gardening or completing “odd jobs” at home. Topic 14 (4.23%; Doing online activities) included participants spending time on activities online, including classes, courses, and group sessions (such as singing groups) as well as functional online activities such as ordering supermarket deliveries.

Amongst the remaining topics, Topic 2 (8.14%; Engaging in harmful behaviours) included individuals who reported self-harming or increasing alcohol consumption or comfort eating, though the latter two were reported in several cases as improving mood (at least in the short term). Topic 16 (4.13%; Avoiding the news) meanwhile included text on individuals actively avoiding coverage on COVID-19 as a coping strategy. Topic 7 (6.94%; Following the rules) referred specifically to attempts to reduce risk by following guidelines (e.g., mask wearing). Finally, Topic 13 (4.49%; Mixture of themes) surfaced exemplar texts that did not contain a clear, consistent theme.

### Topic Proportions and Author Characteristics

**FIGURE 3:**
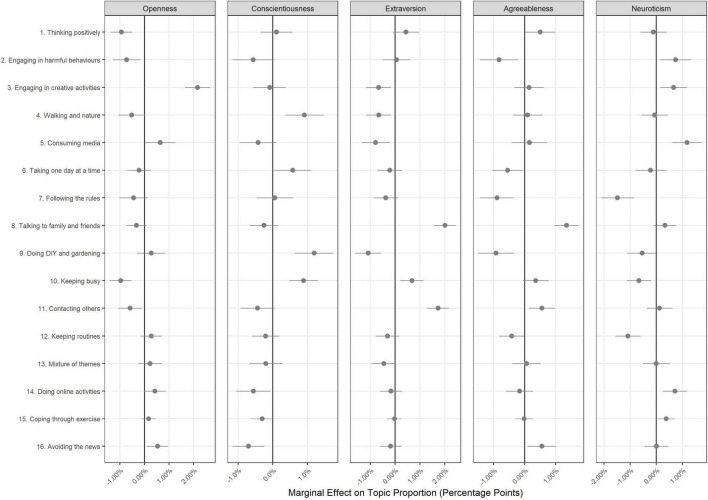
Association between document topic proportion and participant’s Big-5 personality traits (+95% confidence intervals). Results displayed as marginal effects (difference in topic proportion according to change in independent variable). Continuous independent variables are standardized such that a one unit change is equal to a 2 SD difference (Gelman, 2008), Derived from OLS regression models including adjustment for gender, ethnicity, age, education level, living arrangement, psychiatric diagnosis, long-term physical health conditions, self-isolation status, Big-5 personality traits and keyworker status.

**FIGURE 4:**
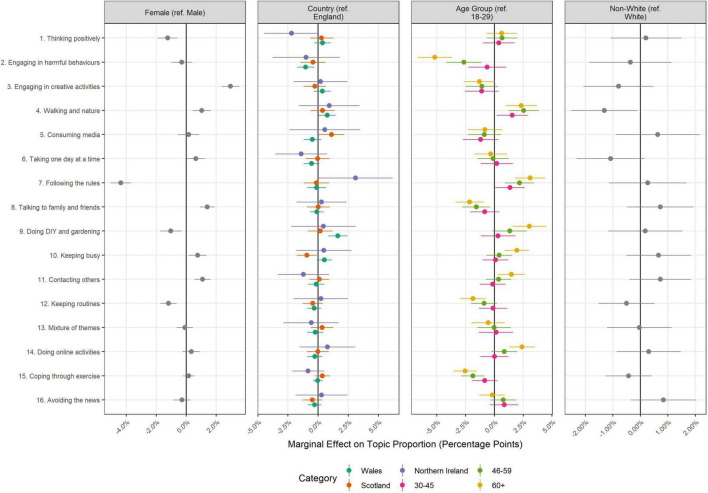
Association between document topic proportion and demographic characteristics (+95% confidence intervals). Results displayed as marginal effects (difference in topic proportion according to change in independent variable). Derived from OLS regression models including adjustment for gender, ethnicity, age, education level, living arrangement, psychiatric diagnosis, long-term physical health conditions, self-isolation status, Big-5 personality traits and keyworker status. Reference categories are provided in the plot titles.

**FIGURE 5:**
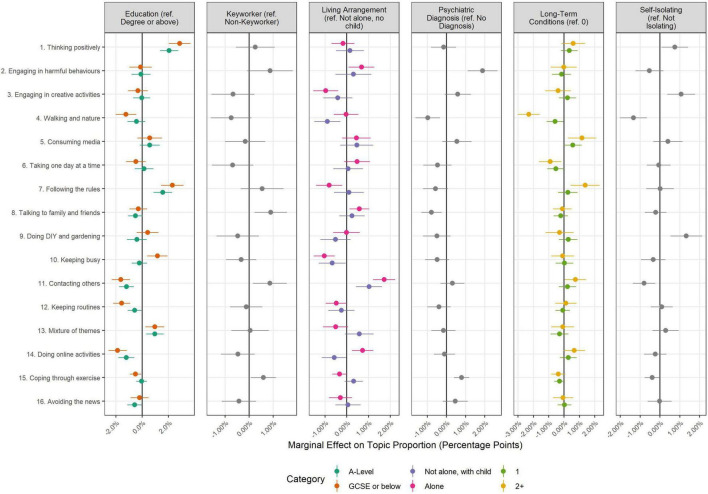
Association between document topic proportion and participants’ socioeconomic and health characteristics (+95% confidence intervals). Results displayed as marginal effects (difference in topic proportion according to change in independent variable). Derived from OLS regression models including adjustment for gender, ethnicity, age, education level, living arrangement, psychiatric diagnosis, long-term physical health conditions, self-isolation status, Big-5 personality traits and keyworker status. Reference categories are provided in the plot titles.

The results of regressions exploring the association between topic proportions and author characteristics are displayed in Figures 3–5. A sizeable number of coefficients were statistically significant when using Bonferroni-corrected *p*-values (*p* < 0.05/352 comparisons; see Supplementary Tables 2, 3 for full regression results). However, effect sizes were generally small.

Regarding Big-5 personality traits (Figure 3), individuals high in trait openness devoted more text to topics such as engaging in creative activities (Topic 3); conscientious individuals devoted more text on keeping busy (Topic 10), walking and spending time in nature (Topic 4), and spending time on DIY or gardening (Topic 9); extravert individuals wrote more on contacting others (Topic 8 and Topic 11) and less on spending time consuming media (Topic 5) or doing DIY or gardening (Topic 9); agreeable individuals devoted more text on avoiding the news (Topic 16), spending time talking to family and friends (Topic 8) and in harmful behaviours (Topic 2); and neurotic individuals wrote more on consuming media (Topic 5) and – surprisingly –less on keeping routines (Topic 12) and following the guidelines (Topic 7). However, associations were small in each case: a 2 SD increase in the relevant trait was associated with a less than 2.5% point difference in proportion of text devoted to a given topic.

There were also differences according to demographic characteristics (sex, country, age, and ethnicity; Figure 4). Some of these differences were relatively sizeable. Notably, females devoted more text to discussing creative activities (Topic 3; 3.0%, 95% CI = 2.4, 3.6%) and less text to discussing following the rules (Topic 7; −4.4%, 95% CI = −5.1, −3.7%). Adults aged 60+ wrote less on engaging in harmful behaviours than adults aged 18–29 (Topic 2; −5.1%, 95% CI = −6.8, −3.5%) and more on following the rules (Topic 7; 3.0%, 95% CI = 1.7, 4.2%) and doing DIY and gardening (Topic 9; 3.1%, 95% CI = 1.7, 4.5%). Differences according to country and ethnicity were generally smaller.

Finally, there were differences according to socio-economic and health characteristics (Figure 5), but effect sizes were less than 3% points in each case. Individuals with degree-level education or above devoted less text to thinking positively (Topic 1) and individuals with psychiatric diagnoses devoted more text to discussing engaging in harmful behaviours (Topic 2; 1.9%, 95% CI = 1.1, 2.7).

### Associations Between Topic Proportions and Lockdown Experiences

**FIGURE 6:**
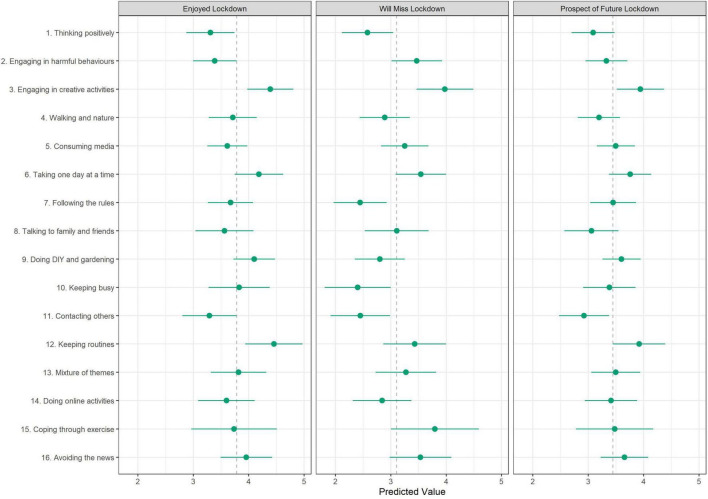
Association between lockdown experiences and document topic proportions (+95% confidence intervals). Results displayed as predicted lockdown experience values where proportion devoted to a given topic is 100%. Derived from OLS regression models adjusting for all estimated proportions for all topics simultaneously. Dashed line represents mean value for the respective lockdown experience variable.

The results of regressions assessing the association between lockdown experiences and topic proportions are displayed in Figure 6. Engaging in creative activities (Topic 3), DIY and gardening (Topic 9) and keeping a routine (Topic 12) were associated with greater enjoyment of first lockdown. Creative activities were also related to feeling more positive (or less negative) about a future lockdown and expecting to miss the first lockdown more. Following the rules (Topic 7), keeping busy (Topic 10), and thinking positively (Topic 1) were related to anticipating missing lockdown less. Talking to family and friends was generally related to worse lockdown experiences (though confidence intervals overlapped mean values).

## Discussion

We identified 16 overarching topics of how people were coping during lockdown in the United Kingdom. The most discussed coping strategy was ‘thinking positively’ and involved themes of gratefulness and positivity. Numerous topics were centered around activities and hobbies including ‘walking and spending time in nature’, ‘coping through exercise’, ‘doing DIY and gardening’, and ‘engaging in creative activities’. Other themes were digitally oriented, including ‘consuming media’ and ‘doing online activities’, or were socially-supportive, including ‘contacting others’ and ‘talking to friends and family’. Other strategies were more focused on staying in control such as ‘keeping routines’, ‘focusing on one day at a time’, ‘keeping busy’, and ‘following government guidelines’. However, some respondents reported adopting more avoidant strategies including ‘engaging in harmful behaviours’ and ‘avoiding the news’.

Many of the core topics we identified echo those found in other coping research conducted during the COVID-19 pandemic, including reports of ‘embracing lockdown’, feeling hope, and the uptake of numerous hobbies and activities (Al-Tammemi et al., 2020; Park et al., 2020; Rogers et al., 2020; Hampshire et al., 2021; Ogueji et al., 2021; Sarah et al., 2021). However, our findings extend some of this previous research by elucidating specific activities within previous broad themes identified (for example, DIY and gardening), and, with regards to previous text mining studies, show that topics identified (e.g., using outdoor space, video-conferencing, keeping routines) were used across a longer time frame that just the beginning of the pandemic (Rogers et al., 2020; Hampshire et al., 2021). In line with previous research, a number of known sociodemographic, personality, and health predictors were associated with coping choice during the pandemic (Park et al., 2020; Ahmed et al., 2021; Fluharty and Fancourt, 2021).

It is tempting when considering coping to attempt to categorise strategies into adaptive vs maladaptive strategies: those that could have supported mental health and experiences during COVID-19 vs. those that exacerbated negative experiences. However, the effectiveness and suitability of coping strategies depends strongly on factors such as the context, the timescale over which the coping strategy is employed, the outcome the strategy is being employed to deal with, whether the strategy occurs in isolation or alongside other strategies, and one’s flexibility to modify their use of the strategy according to situational demands (Folkman and Moskowitz, 2004). Therefore, this study did not attempt to make such simple categorisations. Nevertheless, a number of notable associations between the strategies employed and the experiences of the individuals using them did emerge. For example, it was notable that the largest topic was “thinking positively”. According to Fredrickson’s (2001) broaden and build theory, positive psychological approaches to stressful situations have a critical adaptive purpose to help prepare individuals for future challenges. Positive thinkers typically use problem-focused, functional and efficient coping strategies, which corroborates the findings from this study that thinking positively was associated with taking one day at a time, keeping routines, and keeping busy; all proactive coping strategies (Naseem and Khalid, 2010). By employing such approaches, individuals can appraise stressful situations as less threatening, thereby reducing the potential impact of the external stressful situation (Lazarus and Folkman, 1984). Thus, daily positive emotions can moderate stress reactivity (Ong et al., 2006), and are also associated with a lower risk of developing depression following stressful societal events (Fredrickson et al., 2003). Conversely, negative thinking is associated with coping becoming dysfunctional. This was seen amongst 8% of the sample who reported engaging in harmful behaviours. Notably, these harmful behaviours were most common amongst younger adults (aged 18–29) and people with pre-existing psychiatric diagnoses. This corroborates previous research. For example, there is evidence generally that younger adults are more likely to engage in avoidant coping strategies outside of pandemic circumstances (Hamarat et al., 2002; Chen et al., 2018). But it is notable that these groups have had consistently poorer psychological experiences across the pandemic. This fits with research showing that engaging in avoidant and harmful behaviours is associated with greater psychological reactivity both during the pandemic and outside it (Thompson et al., 2018; Fluharty et al., 2021). Thus data reported here could help to explain some of the coping mechanisms that could underlie such differential experiences.

Additionally, we found that individuals who engaged in creative activities had the most positive lockdown experiences. The association between creative activities and more enjoyable lockdown experiences is consistent with findings that creative activities are beneficial for a range of mental, social, physical, and wellbeing outcomes (Fancourt et al., 2019). The results are also consistent with a recent analysis of the COVID-19 Social Study suggesting that individual’s used arts activities during lockdown as a way to regulate their emotions (Mak et al., 2021) and an analysis that found longitudinal associations between creative activities during lockdowns and improvements in depression, anxiety and well-being (Bu et al., 2021). It is possible that those who were able to engage in creative activities had other advantages, such as higher incomes, larger houses, or fewer caring responsibilities. However, some of the typical barriers to engagement in the arts changed during lockdown as some activities shifted to a virtual platform (e.g., physical attendance, cost effective) (Mak et al., 2021), which may mean that some people had an enjoyable lockdown experience as they were able to participate in activities they would have otherwise been unable to.

There was also an association between engaging in DIY and gardening and more positive experiences during lockdown. This echoes previous work on the longitudinal associations between outdoor activities during lockdowns and improvements in mental health and wellbeing (Bu et al., 2021; Stock et al., 2021). Being outdoors during lockdowns may have helped to remove individuals from stressful home environments and has also been shown to aid recovery from mental exhaustion, increase one’s sense of vitality (physical and mental energy), and increase physical activity, which in turn can support better mental health and coping (Kaplan, 1995; Markevych et al., 2017). However, it is also notable that we did not find associations between some strategies and experiences. For example, in other studies consuming media during COVID-19 has been associated with poorer mental health (Bu et al., 2021), but we did not find any association with people’s enjoyment of lockdown or attitudes towards potential future lockdowns. This supports suggestions that the effects of coping strategies depend on a wide-range of factors including the specific outcomes in question and adds weight to the importance of considering coping during COVID-19 as a complex phenomenon (Folkman and Moskowitz, 2004). Our results also highlighted the importance of socially-supportive strategies during the pandemic. Recent evidence suggests socially-supportive strategies (e.g., talking to friends and family, social media, contacting others) have been the most commonly employed coping strategies during the COVID-19 pandemic (Al-Tammemi et al., 2020; Ogueji et al., 2021; Sarah et al., 2021), and in other infectious disease outbreaks (Chew et al., 2020). Socially-supportive coping during the pandemic has been associated with faster decreased in mental health symptoms during the first lockdown, suggesting it was a particularly effective form of coping (Fluharty et al., 2021). While the most frequently reported strategy in the current study was not socially-supportive (thinking positive), there were two different coping strategies (11.91%) related to a socially-supportive theme (contacting others and talking to friends and family). Previous research has also reported use of such strategies amongst specific populations such as keyworkers and people living alone as ways of maintaining resilience and combatting loneliness, but it was previously unclear whether such usage was above and beyond that of other demographic groups (May et al., 2021). Our results suggest that not only did these groups use such strategies but they were more likely to do so than non-keyworkers and people not living alone.

This study had several strengths. We used rich qualitative data from over 11,000 United Kingdom adults representing a wide range of demographic groups. By using open-ended free-text data, we were able to analyse spontaneous responses and thus were not limited to coping strategies, activities, or styles we had thought of in advance. Some of the coping strategies were related to participant characteristics in the expected direction – for instance, people with pre-existing mental health conditions were more likely to report engaging in harmful behaviours (in line with previous research that this group is more likely to use avoidant coping). This suggests that our models extracted consistent and meaningful themes. While structural topic models are novel in the coping literature, our results show that such models can complement and bridge qualitative and quantitative approaches, providing insights not easily attained with either approach on its own. A further strength of this study was that we used data from 7 to 8 months after the first lockdown, allowing for an assessment of coping strategies across an extended period of the pandemic.

Nevertheless, this study had several limitations. Not all of the topics identified a single theme consistently and associations with participant characteristics could be driven by idiosyncratic texts. Our sample, though heterogeneous, was not representative of the United Kingdom population. Respondents to the free-text question were also biased towards the more highly educated. This may have generated bias in the topic regression results. While it is plausible that participants discussed coping strategies that they deemed most important, participants may have employed multiple coping strategies and not written about them all. Further, across the long timespan of the pandemic, individuals may have adopted different strategies at different points. Responses may have been biased towards those salient at the time (e.g., those used recently). Moreover, individuals may not interpret or be aware of a behaviour as a coping strategy, though it has that effect – for instance, increasing consumption of alcohol or fatty or sugary foods. A final limitation was that, while we included a wide set of predictors in our models, many relevant factors were unobserved. Associations may be biased by unobserved confounding.

## Conclusion

Sixteen different coping strategies employed by adults in the United Kingdom during the COVID-19 pandemic were identified through text-mining participant free test responses to the COVID-19 Social Study. Some strategies reported were more cognitive (or “antecedent-focused”), either based around attentional deployment (both focusing attention onto the pandemic by focusing on following the rules or distracting oneself from events by avoiding the news), problem solving (e.g. drawing on social support) or cognitive change (e.g. trying to think more positively about things) (Gross, 2001). Others were response focused, involving the use of hobbies, exercise or substances to cope. Some coping strategies reported help to explain why certain groups have coped better than others in the pandemic, reporting lower scores of anxiety and depression. This finding may be useful for helping individuals prepare for future lockdowns or other events resulting in self-isolation. Socially supportive coping also emerged as an important coping strategy used by certain groups at higher risk of poor mental health such as keyworkers and people living alone, highlighting the importance of supporting individuals at risk of increased loneliness and lower social support during pandemics to connect with others. However, more research is needed around coping strategies involving potentially harmful and risky behaviours to identify if such behaviours predict poorer mental and physical health during pandemics and how they can be avoided. Overall, this study sheds light onto the important topic of how people adapted to the challenging circumstances of the COVID-19 pandemic and how coping strategies varied by sociodemographic factors. Given that individuals’ roles in pandemics (i.e., survivor, healthcare, patient, caregiver, general population) can also affect how we cope (Chew et al., 2020), future research may want to extend the findings here to explore the interaction between coping strategies, individual roles and subsequent mental health trajectories.

## Data Availability Statement

The datasets presented in this article are not readily available due to stipulations made by the ethics committee. Requests to access the datasets should be directed to LW, liam.wright@ucl.ac.uk.

## Ethics Statement

The studies involving human participants were reviewed and approved by UCL Research Ethics Committee (12467/005). The patients/participants provided their written informed consent to participate in this study.

## Author Contributions

All authors conceived and designed the study. LW curated the data and conducted the data analysis. LW and MF agreed on narrative titles for the topics and wrote the first draft. All authors provided critical revisions, read, and approved the submitted manuscript.

## Conflict of Interest

The authors declare that the research was conducted in the absence of any commercial or financial relationships that could be construed as a potential conflict of interest.

## Publisher’s Note

All claims expressed in this article are solely those of the authors and do not necessarily represent those of their affiliated organizations, or those of the publisher, the editors and the reviewers. Any product that may be evaluated in this article, or claim that may be made by its manufacturer, is not guaranteed or endorsed by the publisher.
